# Synchronous Papillary Carcinoma and Hemangiopericytoma with Lung Metastases

**DOI:** 10.1155/2013/231758

**Published:** 2013-12-04

**Authors:** Nicola Malagutti, Valeria Iannini, Andrea Rocchi, Francesco Stomeo, Antonio Frassoldati, Michela Borin, Stefano Pelucchi

**Affiliations:** ^1^Department of Otolaringology, Azienda Ospedaliero-Universitaria St. Anna di Ferrara, Via A. Moro 8, 44124 Cona (Ferrara), Italy; ^2^Department of Oncology, Azienda Ospedaliero-Universitaria St. Anna di Ferrara, Via A. Moro 8, 44124 Cona (Ferrara), Italy

## Abstract

Hemangiopericytomas (HPC) are uncommon tumors that originate from perivascular cells of capillary vessels. HPC are about 1% of all vascular tumors and can be found in the head-neck region with an incidence between 16% and 33%. HPC is a neoplasm of uncertain malignant potential; it can behave as an aggressive tumor with metastases and increased mitotic activity or as a relatively benign neoplasm with only local development. In this paper we describe a case of hemangiopericytoma with uncertain malignant potential with cervical location associated with a concomitant papillary thyroid carcinoma and lung metastasis of unknown origin; this case led us to follow a specific and uncommon diagnostic and therapeutic strategy.

## 1. Introduction

Hemangiopericytomas (HPC) are uncommon tumors that originate from perivascular cells known as pericytes of Zimmerman, localized around capillary vessels. For the first time these neoplasms were called hemangiopericytomas in 1942 by Stout and Murray who described them as tumors composed of proliferation of capillary vessels and rounded cellular elements [1].

Pericytes have characteristics of smooth muscle cells and probably have role in blood flow regulation. After studies with electronic microscope, it has been supposed that these cells could be derived from primitive mesenchymal precursors and could represent a form of transition between mesenchymal and smooth muscle cells [2].

Hemangiopericytomas are about 1% of all vascular tumors and can be found in the head-neck region with an incidence between 16% and 33%; HPC have been described inside orbit, nose, oral cavity, parotid gland, parapharyngeal space, jugular foramen, and so forth [3, 4]. This neoplasm can appear at any age but it is predominant between 6th and 7th decade and equally distributed between sexes. Etiology is unknown, but traumas, long therapies with corticosteroids, or hormonal imbalances may be involved [4].

HPC is a neoplasm of uncertain malignant potential; it can behave as an aggressive tumor with metastases and increased mitotic activity or as a relatively benign neoplasm with only local development [5].

Immunohistochemical profile is often heterogeneous with characteristics close to solitary fibrous tumors.

HPC are classified as benign, borderline, and malignant, depending on some histopathological features (mitotic activity, cellularity, and nuclear atypia) and clinical behavior (presence of necrosis and tumor size) [6, 7]. However, the histopathological distinction between benign and malignant HPC can be difficult. In fact, the biological behavior of these tumors is quite particular: some cases of HPC with benign immunohistochemical characteristics and low mitotic index can appear with distant metastasis and bad prognosis. Due to the unpredictable clinical and biological behavior, radical surgical excision is recommended when possible.

We describe a case of cervical haemangiopericytoma with uncertain malignant potential associated with papillary thyroid carcinoma and unclear lung metastasis.

## 2. Case Report

A 63-year-old Caucasian woman came to our otolaryngology department complaining of a right lateral neck swelling since three months. The patient did not have any pain or any other symptoms. Echography revealed the presence of a solid round neoplasm of 45 × 27 mm at II and III neck level; neoplasm was not homogeneously hypoechogenic with presence of unechogenic areas; moreover, echotomography revealed the presence of multiple thyroid nodularities at both sides with maximum size of 16 mm at right and 20 mm at left thyroid's lobe.

**Figure 1:**
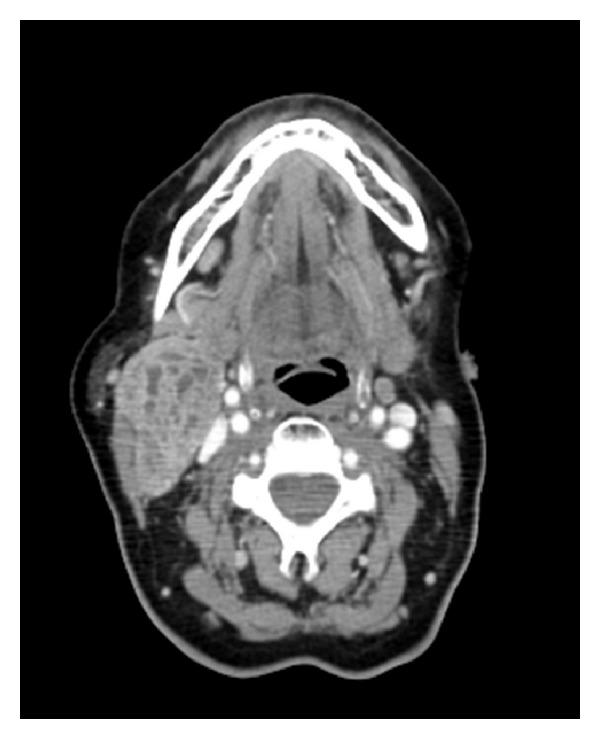
The neoplasm in lateral cervical region (maximum length of 6 cm) confirmed by CT scan.

**Figure 2:**
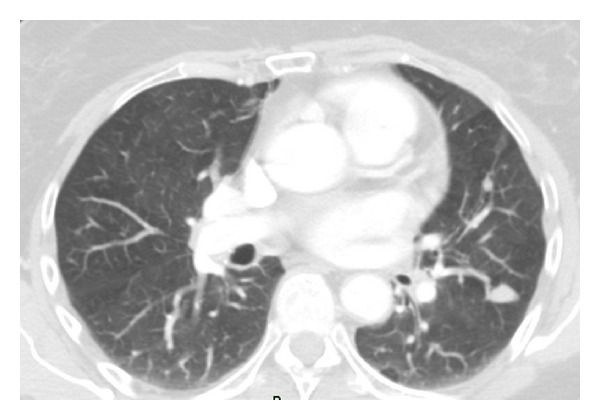
CT scan with lung metastases.

We performed an echo-guided fine-needle aspiration: thyroid's cytology suggested follicular, hyperplastic, and cystic nodules; cervical neoplasm's cytology described cell aggregates with epithelial type morphology and moderate nuclear atypia with some stromal branches. We further made a neck and thorax CT scan that confirmed the presence of a solid neoplasm in right lateral cervical region with maximum length of 6 cm that took inhomogeneous contrast (Figure 1); CT scan even described thyroid's nodular aspects and lung lesions in the superior left lung lobe (15 mm × 12 mm) and in the lower right lung lobe (10 mm) (Figure 2); these findings were radiologically suggestive for lung metastasis but none of them could be reachable by a bronchial or a transthoracic biopsy.

We performed surgical excision of neck neoplasm; during dissection the lesion appeared adherent to the lower pole of the parotid gland and reached II and III neck level; we even performed right superficial parotidectomy and lateral neck dissection levels II and III. Histological examination of the specimen revealed a solitary fibrous hemangiopericytoma of uncertain malignant potential, with moderate nuclear pleomorphism, absence of coagulative necrosis, and mitotic active sites up to 7-8/10 HPF (MIB1 approximately 10%), free from salivary gland tissue lesions (phenotypic characterization: Actin−/CD31−/CD34+/CD99+/CK(PAN)−/CK(HMW)−/Desmina−/c-kit (CD117−/DOG1−/EMA−/S100−/Bcl2+/Ki67 (MIB-1) 10%)); any removed lymph node was affected by metastases.

According to the limited number of metastatic hemangiopericytomas described in the literature we focused on thyroid lesions to look for a primary tumor potentially responsible for pulmonary metastases.

Therefore, we planned a total thyroidectomy with neck dissection of the VI level; histological thyroid's examination revealed the presence of a multifocal and bilateral papillary carcinoma with oncocitary aspects, locally infiltrating thyroid's capsule in the right lobe with absence of lymph node metastases.

To treat thyroid tumor and in order to check lung's metastases behavior, the patient underwent a metabolic radiotherapy with iodine-131 (1870 MBq, 50.6 mCi) under TSH stimulation. After therapy the patient showed a TSH of 29.09 micro IU/mL, a HTG of 0.8 ng/mL, and a AbTg of 174 IU/mL.

After 8 days scintigraphy revealed a focal radioiodine concentration in the thyroid's region but lung lesions did not show any iodine- 131 uptake.

The patient was controlled after 4 months from thyroidectomy with neck and chest CT scan; the exam confirmed the presence of the same lung lesions already described that kept the same shape and size.

Multidisciplinary evaluation with oncologists, thoracic surgeons, and radiotherapists was then performed; considering difficulties and risks of lung biopsy and radiological stability of metastases and due to the lack of any effective surgical treatment or chemotherapy even in case of confirmed metastatic hemangiopericytoma, we decided to proceed simply by following up the patient for any clinical and radiological evolution of the pathology.

## 3. Discussion and Conclusion

Usually hemangiopericytomas present as a painless swelling with a slow growth tendency so symptoms are often related to compression of other structures. Criteria of malignancy include tumor size (>50 mm), the stage of disease at presentation, infiltration of the margins, high cellularity, nuclear pleomorphism on the areas of tumor necrosis, and the mitotic index [8].

Survival rate is then correlated with degree, size, and margin status. Enzinger and Smith [9] analyzed 106 cases of hemangiopericytoma and in their study, 16% of patients had tumor localization in the head and neck, and total survivors were 70%. The authors defined a high-grade lesion if it had more than four mitotic figures per high-power field, if it showed a high cellularity or necrosis.

In another analysis of 45 cases of HPC of the head and neck 40% had local recurrence and 19% showed distant metastases [10].

Metastases may occur in lungs, bones, liver, regional lymph nodes, and pancreas [11].

Treatment of choice for each location is excision, where it is possible. Efficacy of adjuvant radiation therapy is not homogeneously supported by literature although many recent studies suggest that it may be useful when surgical removal of the tumor is incomplete [12]. External radiotherapy has even been used as adjuvant therapy for the treatment of local recurrences after surgical resection. At a fractionated focal dose of 50 Gy, studies have shown a significant increase of the recurrence-free period [13].

The role of chemotherapy in the treatment of HPC is still not clear: it can be used before surgery to reduce tumor's size, for postoperative metastasis as adjuvant, and for unresectable HPC as palliative therapy [14].

Several chemotherapeutic agents have been used for treatment of unresectable or metastatic HCP but the choice of drug, dose, method of administration, schema, or response criteria is still discussed. Adriamycin, alone or in combination, seems to be the most effective agent obtaining complete or partial remissions in 50% of treated cases. Other drugs, such as cyclophosphamide, vincristine, methotrexate, actinomycin, and DTIC, may have some antitumor activity, but due to the small number of patients treated, it is difficult to determine the best combination of these drugs [15, 16].

Recently some cases of patients treated with antiangiogenic drugs have been described, in particular with sunitinib, sorafenib, and dasatinib [17–19].

Sensitivity of HPC to inhibitors of angiogenesis is maybe due to the high vascular component and to the expression of platelet-derived growth factor receptor (PDGFR) and vascular endothelial growth factor receptor (VEGFR).

In the case we reported, the association of thyroid's papillary carcinoma and HPC led us to follow a specific and uncommon diagnostic and therapeutic strategy; the slow growth of lung metastases and the limited efficacy of currently available treatments induced us to avoid invasive lung biopsy or chemotherapy. Nowadays we still follow up this patient: up to now, 2 years after diagnosis, at CT scan lung metastases of HPC are still of the same size.
